# Structure, function, and research progress of primary cilia in reproductive physiology and reproductive diseases

**DOI:** 10.3389/fcell.2024.1418928

**Published:** 2024-06-03

**Authors:** Xiaochuan Long, Li Chen, Xinyao Xiao, Xiayu Min, Yao Wu, Zengming Yang, Xin Wen

**Affiliations:** ^1^ Clinical Veterinary Laboratory, College of Animal Science, Guizhou University, Guizhou, China; ^2^ Key Laboratory of Animal Genetic, Breeding and Reproduction in the plateau Mountainous Region, Ministry of Education, Guizhou University, Guizhou, China; ^3^ Basic Veterinary Laboratory, College of Animal Science, Guizhou University, Guizhou, China

**Keywords:** primary cilia, reproductive physiology, ciliary diseases, Hh signaling pathway, Wnt signaling pathway

## Abstract

Primary cilia, serving as the central hub for cellular signal transduction, possess the remarkable ability to translate diverse extracellular signals, both chemical and mechanical, into intracellular responses. Their ubiquitous presence in the reproductive system underscores their pivotal roles in various cellular processes including development, differentiation, and migration. Emerging evidence suggests primary cilia as key players in reproductive physiology and associated pathologies. Notably, primary cilia have been identified in granulosa cells within mouse ovaries and uterine stromal cells, and perturbations in their structure and function have been implicated in a spectrum of reproductive dysfunctions and ciliary-related diseases. Furthermore, disruptions in primary cilia-mediated signal transduction pathways under pathological conditions exacerbate the onset and progression of reproductive disorders. This review provides a comprehensive overview of current research progress on primary cilia and their associated signaling pathways in reproductive physiology and diseases, with the aim of furnishing theoretical groundwork for the prevention and management of primary cilia-related structural and functional abnormalities contributing to reproductive system pathologies.

## 1 Introduction

Primary cilia, resembling hair-like structures situated on the cell membrane surface and originating from the centrosome, possess the capacity to detect diverse extracellular stimuli. Recent investigations have revealed the intimate association between primary cilia and diverse intracellular signal transduction mechanisms, often likened to “cellular antennas” and “signal amplification receptors” (Patnaik et al., 2020). Despite being stationary, primary cilia possess a ciliary membrane capable of sensing chemical and mechanical signals from the surrounding environment (Barnes et al., 2021). This membrane harbors a plethora of signal receptors and ion channels, facilitating the conversion of extracellular signals into intracellular signals for cascading, a process critical for intercellular signal transduction. Numerous signaling pathways are mediated by primary cilia, encompassing Hedgehog, Wnt, mTOR, GPCR, Notch, Hippo, and TGF-beta signaling pathways (Pala et al., 2017; Anvarian et al., 2019). Structural and functional aberrations in primary cilia can detrimentally affect the functionality of associated signaling pathways, precipitating a spectrum of ciliary-related disorders (Ng et al., 2021), including recurrent implantation failure (RIF), epithelial ovarian cancer (EOC), breast cancer, prostate cancer (PCa), as well as autosomal recessive genetic disorders such as Bardet-Biedl syndrome and Joubert syndrome.

Research indicates that the growth and development of the mammalian reproductive system are governed by a multitude of signaling pathways, including Hedgehog, Wnt, and mTOR. Dysregulated signal transduction may result in developmental anomalies of embryonic organs, including ovarian development and uterine decidualization (Anvarian et al., 2019). This paper presents a comprehensive review of pertinent primary cilia and their correlated signaling pathways within the reproductive system, with the goal of furnishing theoretical insights for the prevention and treatment of reproductive system disorders stemming from primary ciliary dysfunctions.

## 2 Structure and function of primary cilia

**FIGURE 1:**
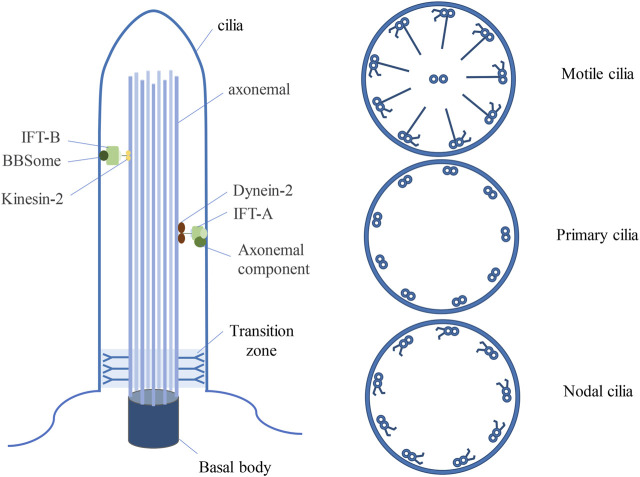
Three types of ciliated structures. Cilia, resembling hair-like protrusions, emerge from the cell membrane, originating from the centrosome. Motile cilia exhibit a 9 + 2 architecture, comprising nine doublets of microtubules alongside a central pair, complemented by dynein arms. Primary cilia feature a 9 + 0 arrangement, characterized by solely nine peripheral microtubule doublets, devoid of central microtubules and dynein arms. Nodal cilia display a 9 + 0 configuration, showcasing nine pairs of peripheral microtubules, devoid of a central pair yet equipped with dynein arms. BBSome is a multisubunit protein complex that is not necessary for the maintenance of ciliary integrity, but is an important determinant of ciliary protein composition.

Cilia are ubiquitous organelles in mammalian cells, being present in nearly all cell types. Previous research has delineated several types of cilia, encompassing motile cilia, primary cilia, and nodal cilia (Satir and Christensen, 2008) (Figure 1). Motile cilia consist of 9 doublet microtubules encircling a pair of central single microtubules, thereby constructing a 9 + 2 axoneme (Hua and Ferland, 2018). These organelles are restricted to specific cell types and exhibit motility. Conversely, primary cilia are generally found on various cell types, typically manifesting as solitary structures protruding from the cell surface, where they serve as pivotal “signal enhancers” in intercellular signal transduction. (Berbari et al., 2009). In contrast to motile cilia, primary cilia possess a 9 + 0 axoneme structure, devoid of the central microtubule pair and dynein arms characteristic of motile cilia (Li, 2022). Nonetheless, there are exceptions to this classification, exemplified by olfactory cilia, categorized as primary cilia despite featuring a 9 + 2 axoneme configuration devoid of dynein arms (Perry et al., 2009). Nodal cilia emerge during embryonic development, exhibiting a primary cilia 9 + 0 axoneme structure alongside the dynein arms configuration characteristic of motile cilia (Hua and Ferland, 2018).

Primary cilia consist of microtubule axonemes, basal bodies, transition zones, and transition fibers. They exhibit diminutive dimensions, boasting a diameter of approximately 0.2 μm and a length spanning from 3 to 10 μm. Primary cilia, microstructures anchored by the centrosome, conspicuously extend from the surface of the majority of eukaryotic cells. The formation of primary cilia is intricately linked to the centrosome. Serving as the microtubule organizing center, the centrosome assumes a pivotal role in orchestrating microtubule dynamics and cell division (Plessner et al., 2019). Primary cilia formation is accomplished through the centrosome’s specific functions during distinct stages of the cell cycle (Luan et al., 2021). Throughout mitosis, cilia undergo disassembly to permit centrosomal assembly of the spindle apparatus (Jeffries et al., 2019). Upon completion of mitosis and subsequent re-entry into the G0 or G1 phase, centrosomal microtubules extend towards the tip, giving rise to the axoneme, the primary structural feature of cilia (Maskey et al., 2015; Miyamoto and Matsuura, 2015). At this juncture, the centrosome persists in its attachment to the cell membrane at the basal body, undergoing transition into the basal body, and initiating the assembly of nascent cilia (Abreu and Dantas, 2021). Upon re-entry into the cell cycle, the ciliary axoneme undergoes reabsorption, and the basal body ceases to furnish structural support for the cilia (Ford et al., 2018). The transition zone and transition fibers serve as barriers, impeding the free diffusion of large proteins into and out of the primary cilium (Wachten and Mick, 2021). The BBSome, a multi-subunit protein complex, serves as a central mediator facilitating transport across the transition zone (Goetz et al., 2017). Although not indispensable for the maintenance of ciliary integrity, the BBSome plays a pivotal role in determining the composition of ciliary proteins (Wachten and Mick, 2021). Furthermore, primary cilia membranes are adorned with myriad signaling pathway receptors, adept at sensing stimuli from the extracellular milieu (Ortizcruz et al., 2021). Disruptions in the signaling pathways of primary cilia may result in developmental and physiological deficiencies within the organism (Ma et al., 2022).

**FIGURE 2:**
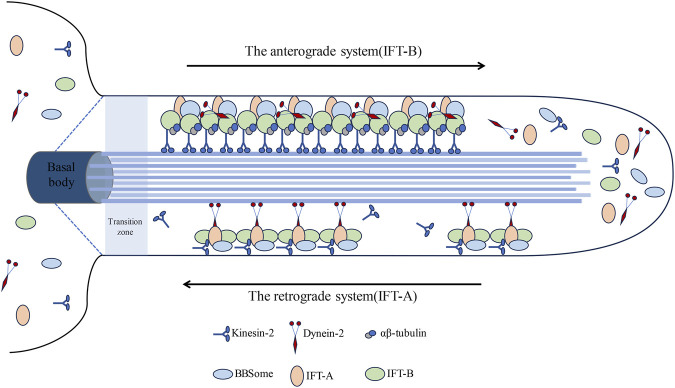
Visually represents the intricate architecture of the intraflagellar transport (IFT) system within cilia. The intraflagellar transport (IFT) system of primary cilia comprises two distinct components: IFT-A and IFT-B. Initially, IFT is recruited to assemble protein complexes at the base of the cilium. IFT-B constitutes a chiefly anterograde system, powered by kinesin-2, facilitating the transportation of cargo synthesized at the ciliary base (basal body) across the transition zone to the tip; conversely, IFT-A operates as a primarily retrograde system propelled by dynein-2, whereupon they reassemble into retrograde trains, conveying their cargos back to the basal body for recycling.

Primary cilia, serving as antennae, have the capability to sense the cellular microenvironment and play a pivotal role in mediating the signal transduction of diverse signaling pathways (Zhang et al., 2023). Within primary cilia resides a highly conserved bidirectional transport system known as the Intra-Flagellar Transport (IFT) system, initially identified in Chlamydomonas (Prevo et al., 2017). The IFT system, functioning as an intracellular transport system, is propelled by kinesin-2 and cytoplasmic dynein-2 (24) (Figure 2). This system operates bidirectionally, employing motor and driving proteins for anterograde and retrograde transport, correspondingly (Seeley et al., 2009).

The anterograde system (IFT-B) comprises 16 proteins (IFT20, IFT22, IFT25, IFT27, IFT38, IFT46, IFT52, IFT54, IFT56, IFT57, IFT70, IFT74, IFT80, IFT81, IFT88, IFT172), facilitating the transportation of proteins to the cilium’s distal tip (Lacey et al., 2023). The retrograde system (IFT-A) shuttles proteins back to the cilium’s base and primarily comprises six proteins (IFT43, IFT121, IFT122, IFT139, IFT144, IFT140) (Li, 2022; Lian et al., 2022). This bidirectional transport mechanism facilitates the regulated growth, maintenance, and disassembly of cilia (Wang et al., 2021; Shi et al., 2023). ADP-ribosylation factor-like protein 13B Arl13b and Intraflagellar Transport protein IFT88 are pivotal components in cilium assembly and play indispensable roles in Hedgehog (Hh) signal transduction (He et al., 2018; Shi et al., 2023; Fitzsimons et al., 2024). Moreover, histone deacetylase HDAC6 and Aurora kinase A are essential for the disassembly of cilia (Xiang et al., 2017; Rowson et al., 2018; Zhang et al., 2021). Previous experimental findings have demonstrated that inhibiting IFT-A proteins markedly shortens primary cilia (Piperno et al., 1998; Iomini et al., 2009), whereas inhibition of IFT-B proteins impedes primary cilia formation (Brazelton et al., 2001; Hou et al., 2007).

The intraflagellar transport (IFT) complex, primarily constituted by IFT-A and IFT-B subcomplexes, assumes a pivotal role in the assembly and upkeep of cilia. The IFT complex encompasses no fewer than 20 varieties of IFT proteins, where IFT-A predominantly encompasses core subunits (IFT122, IFT140, IFT144) alongside peripheral subunits (IFT43, IFT121, IFT139). IFT122 assumes a crucial role within the IFT-A complex, fostering connections between the IFT-A core and peripheral subcomplexes via interactions with IFT43-IFT121 dimers (Takahara et al., 2018). Mutations leading to malfunctioning IFT122 are linked to cranioectodermal dysplasia diseases, which hinder ciliary protein transport but not ciliogenesis (Takahara et al., 2018). IFT43 emerges as the smallest protein within the IFT-A assembly, whereas other constituents of the IFT-A complex, except for IFT43, manifest as sizable proteins with molecular weights surpassing 120 kDa (Taschner et al., 2012). Core constituents of the IFT-B complex, such as IFT88, IFT81, IFT74, IFT52, IFT46, IFT27, IFT70, IFT25, and IFT22, engage in interactions with peripheral proteins (IFT172, IFT80, IFT57, IFT54, IFT20). Among the IFT proteins, IFT172 emerges as the largest (Wang et al., 2018), juxtaposed with IFT20, which represents the smallest counterpart (Yuan et al., 2014). Functioning as a core constituent of the IFT-B complex, IFT52 assumes a pivotal responsibility in upholding the structural integrity of the entire assembly and expediting cargo movement along the ciliary axoneme, thereby implicating its involvement in a spectrum of ciliopathies (Udupa and Ghosh, 2024). Peripheral protein assemblies of IFT-B exert pivotal functions in regulating IFT processes and orchestrating targeted vesicle trafficking from the Golgi network to the ciliary pocket (Lee et al., 2018). Sustained interactions between the IFT-A and IFT-B complexes stand as indispensable for facilitating GPCR-mediated retrograde transport of ciliary proteins (Lee et al., 2018; Kobayashi et al., 2021). Besides the IFT complex, the BBS multi-subunit complex also participates in the transportation of membrane proteins to cilia (Jin et al., 2010), whereby the IFT-A and -B subcomplexes coalesce through the BBSome multi-subunit complex (Nachury et al., 2007).

Research has demonstrated that deficiencies in IFT20, IFT52, IFT80, and IFT88 during mouse limb development can result in skeletal abnormalities (Kitami et al., 2019; Chinipardaz et al., 2022; Guleria et al., 2022; Yamaguchi et al., 2022; Chen and He, 2023). Moreover, mutations in IFT25, IFT27 interfere with the Hedgehog signaling pathway (Keady et al., 2012; Eguether et al., 2014; Yang et al., 2015; Ge et al., 2021), while silencing of IFT80 diminishes Hedgehog signaling but enhances Wnt signaling, underscoring the ability of primary cilia to modulate Hedgehog and Wnt signaling for the regulation of cartilage development (Yang and Wang, 2012; Wang et al., 2013). IFT122 is capable of governing mouse embryo palatal mesenchymal cells (mEPMCs) through the Sonic Hedgehog (SHH) signaling pathway mediated by primary cilia, with silencing of IFT122 leading to impairments in primary cilia growth in mEPMCs (Guo, 2020). Mutations in TALPID3 result in the loss of primary cilia and hinder Hedgehog signal transduction (Yin et al., 2009; Ben et al., 2011; Liu et al., 2021). Therefore, deficiencies in any IFT proteins may lead to structural and functional abnormalities in primary cilia and associated signaling pathways, culminating in the onset of cilia-related diseases.

## 3 Related signaling pathways

### 3.1 Hedgehog signaling pathway

The Hedgehog (Hh) signaling pathway stands as the most thoroughly investigated signaling cascade associated with primary cilia. This pathway is essential not only for embryonic development and organogenesis but also for the maintenance and repair of adult tissue homeostasis (Chen et al., 2023). Anomalous activation of the Hh pathway is pivotal for the pathogenesis of various cancers, encompassing medulloblastoma, basal cell carcinoma, breast cancer, prostate cancer, melanoma, lung cancer, and pancreatic cancer (Ross et al., 2005; Luu et al., 2009; Seeley et al., 2009; Yuan et al., 2010; Kim et al., 2011; Basten and Giles, 2013; Gradilone et al., 2013; Hassounah et al., 2013; Wang, 2021; Giammona et al., 2023). In mammals, Hh signal transduction is orchestrated through primary cilia located on cells (Li et al., 2022). Recognized Hh signaling pathways comprise Indian Hedgehog (Ihh), Desert Hedgehog (Dhh), and Sonic Hedgehog (Shh) (Lienkamp et al., 2012; Feltran et al., 2024). Ihh is the main mediator of progesterone signaling in the mouse uterus and is essential for mediating the interaction between the uterine epithelium and stroma required for embryo implantation (Matsumoto et al., 2002; Lee et al., 2006). Dhh is primarily expressed in the reproductive glands. The Shh signaling pathway serves as a pivotal regulator of early embryonic development, albeit its regulatory mechanisms remain incompletely elucidated (Wang et al., 2016a).

**FIGURE 3:**
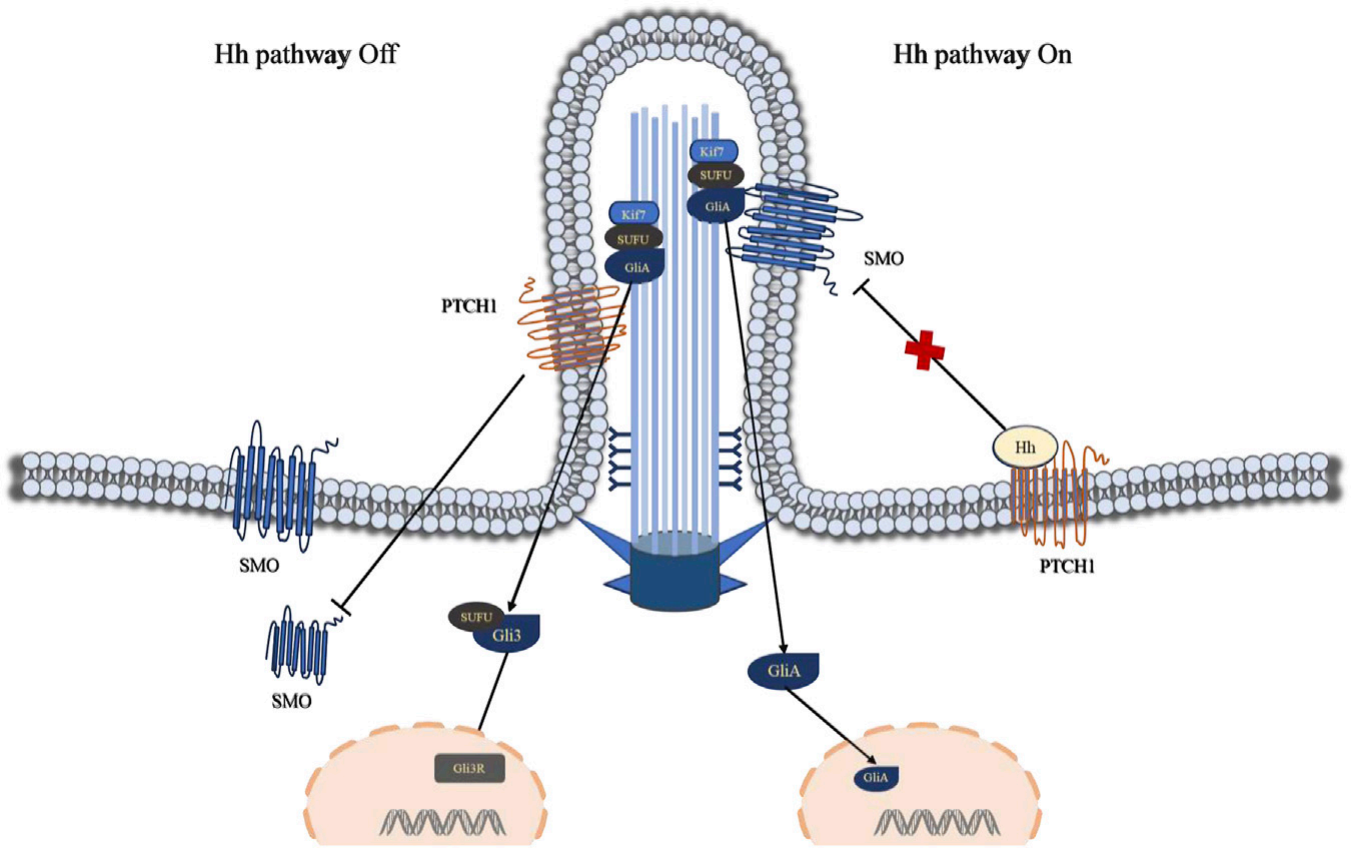
Visually portrays the Hedgehog (Hh) signaling pathway mediated by primary cilia. Ptch1 plays a pivotal role in the primary cilium Hh signaling pathway, exerting its ability to suppress the accumulation of the transmembrane protein Smo within the primary cilium. At this juncture, the transcription factor Gli resides at the cilium’s tip, rendered inactive, while the Hh signaling pathway remains dormant. The interaction between the Hh ligand and Ptch1 triggers Ptch1 to exit the cilium and accumulates in non-ciliary membrane (vesicles or plasma membrane), resulting in the accumulation of Smo within the cilium and the translocation of the transcription factor Gli from the cilium’s tip to the cell nucleus, consequently activating the Hh pathway. Abbreviations: Hh, Hedgehog; SMO, Smoothened; PTCH1, Patched 1; Kif7, kinesin family member 7; SUFU, suppressor of fused; Gli3, Glioma-associated oncogene 3.

The Hedgehog (Hh) signaling pathway encompasses secreted glycoprotein ligands (Hh), two principal membrane protein receptors (Ptch and Smo), fusion inhibitory protein (Su Fu), transcription factor glioma-associated oncogene homolog (Gli), and downstream target genes (Xia et al., 2016). Gli stands as the central element of the Hh signaling pathway, comprising three GLI family members: Gli1, Gli2, and Gli3. Moreover, Ptch, Smo, SuFu, and Gli proteins are localized within primary cilia (Chen et al., 2009). Ptch1 has the ability to impede the accumulation of transmembrane protein Smo within primary cilia (Weiss et al., 2019). At this juncture, the transcription factor Gli resides at the cilium’s distal end, remaining inactive, thereby closing off the Hedgehog signaling pathway. Studies have demonstrated that the secreted glycoprotein ligand Hh interacts with the transmembrane protein receptor Ptch1 on the cilium membrane, prompting Ptch1 to exit the cilium and translocate into the cytoplasm. Consequently, Smo accumulates within the cilium, prompting the transcription factor Gli to translocate from the cilium’s distal tip to the nucleus, thereby initiating the activation of the Hedgehog pathway (Figure 3).

Research has demonstrated that disrupting the IFT25 gene in mice leads to diminished IFT function or disruption of Hedgehog (Hh) signaling pathway transduction ((Ge et al., 2021; Werner et al., 2015)). Mutations in the DHH gene have been linked to male gonadal dysgenesis and the development of spermatocytic tumors (Werner et al., 2015; Sato et al., 2017). In vertebrates, primary cilia serve a crucial function in preserving the integrity of Hedgehog signaling pathways, and alterations in their structure and function can impede the signal transduction process of the Hedgehog pathway (Higgins et al., 2019). The Hedgehog signaling pathway represents a pivotal signaling cascade that governs the development of the male reproductive system (including testicular development, steroidogenesis, and spermatogenesis) (Wang, 2009; O Hara et al., 2011).

Primary cilia act as central hubs for signal transduction, orchestrating the transmission of molecular signals, although the precise molecular regulatory mechanisms remain incompletely elucidated. Mounting evidence indicates that dysregulated activation of the Hedgehog pathway correlates with the onset of diverse tumor types and fosters cancer cell proliferation, metastasis, and the preservation of cancer stem cells. Furthermore, investigations have revealed that Hedgehog signaling crosstalks with other signaling pathways, including Wnt, mTOR, and Notch, albeit the precise regulatory mechanisms remain elusive.

### 3.2 Wnt signaling pathway

The Wnt signaling pathway serves as a primary regulator of cell polarity, cell development, and the preservation of cellular homeostasis. Thus far, Wnt proteins and 10 Frizzled (FZD) receptors have been identified in mammals (Lee, 2020). Depending on the participation of β-catenin, Wnt signaling transduction can be categorized into two branches: the canonical and non-canonical Wnt signaling pathways. Relevant research findings have elucidated an intimate association between primary cilia and the classical Wnt/β-catenin signaling pathway transduction (Zhang et al., 2015). Glycogen synthase kinase-3β (GSK-3β) and Adenomatous polyposis coli (APC), crucial components of the Wnt/β-catenin signaling pathway, are both localized on primary cilia (Wilson and Lefebvre, 2004; Corbit et al., 2008). The canonical Wnt signaling pathway is associated with cell proliferation and differentiation (Moon et al., 2018). In the presence of β-catenin, the Wnt signaling pathway engages Wnt protein with the Frizzled (FZD) receptor and low-density lipoprotein receptor-related protein 5/6 (LRP5/6) (Ma et al., 2022). The scaffold protein Disheveled (Dvl) is recruited to phosphorylated LRP6 on the plasma membrane. Early studies have demonstrated that primary cilia constrain canonical Wnt signaling transduction (Kawata et al., 2021). The presence of primary cilia markedly restricts canonical Wnt signaling transduction in mouse embryo fibroblasts (MEF) and embryonic stem cells cultured *in vitro* (Corbit et al., 2008; Lian et al., 2022).

**FIGURE 4:**
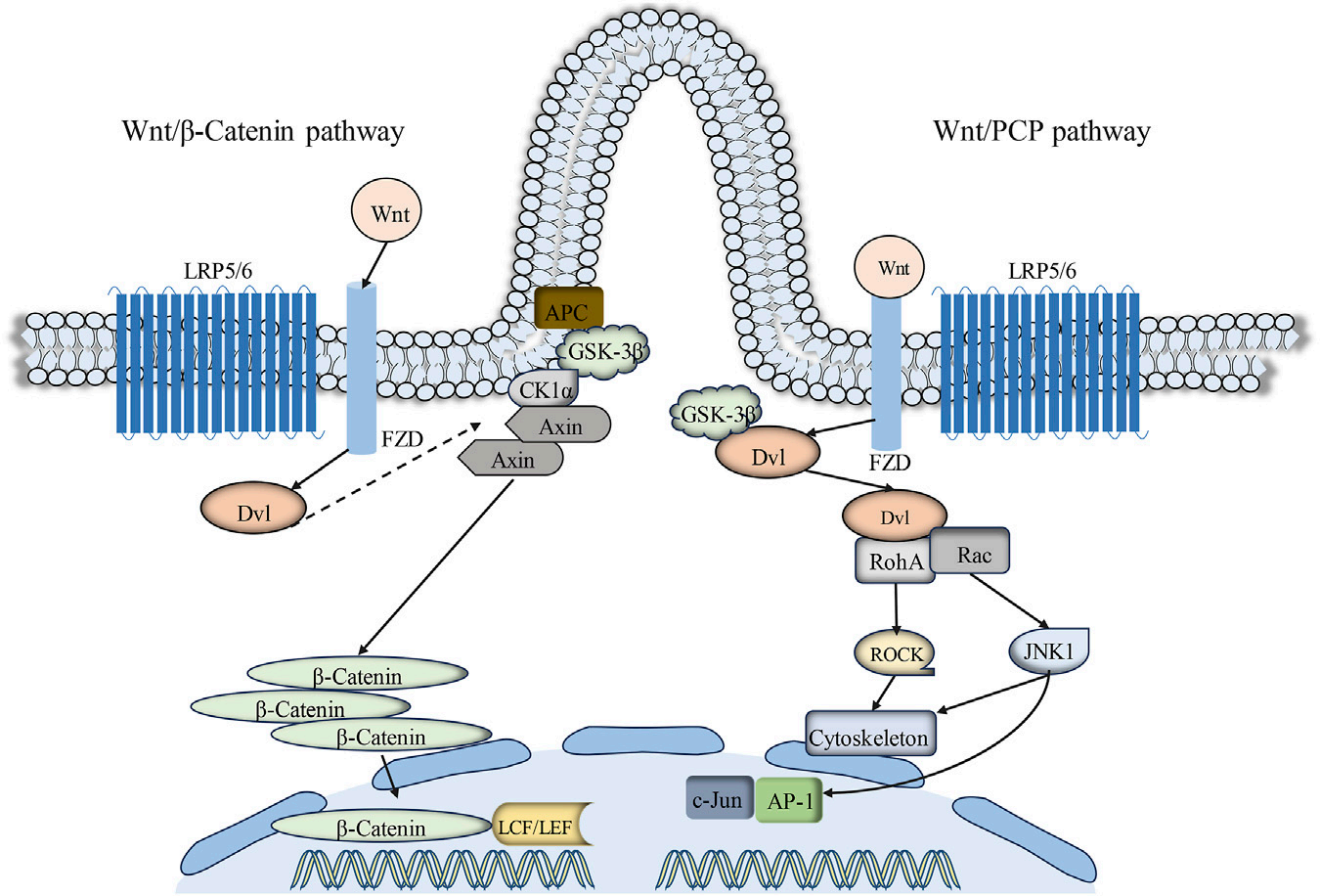
Primary cilia-mediated Wnt signaling pathway. Extracellular Wnt signals engage with the cell surface co-receptors FZD and LRP5/6. Subsequently, the phosphorylation of LRP5/6 and the subsequent signal transduction resulting from DVL and AXIN recruitment to the Wnt-binding receptor collectively contribute to the inhibition of GSK3β activity. This inhibitory process prevents the phosphorylation and degradation of β-catenin, resulting in its cytoplasmic accumulation and subsequent translocation to the cell nucleus. Within the nucleus, β-catenin interacts with TCF/LEF transcription factors, thereby activating Wnt target genes. Conversely, the hallmark of the Wnt/PCP signaling pathway lies in the binding of Wnt ligands to Frizzled receptors, which subsequently activate Disheveled (Dvl) and downstream effectors (such as RhoA and Rac). Abbreviations: FZD, Frizzled; APC, adenomatous polyposis coli; DVL, Dishevelled; AXIN, axis inhibition protein; GSK3β, glycogen synthase kinase three beta; CK1α, casein kinase one alpha; LRP5/6, low-density lipoprotein receptor–related protein 5/6; LEF, lymphoid enhancer-binding factor; TCF, T cell factor; RhoA, Ras homolog family member A; ROCK, Rho-associated protein kinase; JNK1, c-jun N-terminal kinase 1; AP-1, Activator protein-1; Rac, Rac Family Small GTPase.

The Wnt/PCP pathway represents a non-canonical branch of Wnt signaling intimately associated with the regulation of cell polarity, alternatively referred to as the planar cell polarity (PCP) signaling pathway (Zhang et al., 2019; Lee, 2020). Primary cilia are regarded as switches between the canonical and non-canonical branches of the Wnt signaling pathways. The PCP pathway operates independently of β-catenin but relies on Disheveled (Dvl), with ciliogenesis regulated by this pathway (Pruller et al., 2022). Research has identified PCP pathway-associated proteins, including Inversin (NPHP2), Diversin, Vangl-2, and Fat4, which are localized on primary cilia or basal bodies (Morgan et al., 2002; Ross et al., 2005; Saburi et al., 2008; Yasunaga et al., 2011). The association between primary cilia and Wnt signaling transduction has been postulated following the identification of Inversin (Corbit et al., 2008). Inversin interacts with the central molecule of Wnt/PCP signaling transduction, Disheveled (Dvl). Reduction in the expression of the primary cilia protein Kif3a can result in phosphorylation of Dvl (Corbit et al., 2008; Jiang et al., 2016). Moreover, the deficiency of various primary cilia-associated genes (BBS, IFT88, Kif3a, Ofd1) can result in aberrant activation of the canonical Wnt signaling pathway (Chang and Serra, 2013; Liu et al., 2014; Wang et al., 2016b; Yuan et al., 2017) (Figure 4).

Nonetheless, research has indicated that dysfunctional primary cilia do not impact Wnt signaling transduction in cell cultures, fish, or mice. Consequently, the involvement of primary cilia in Wnt signaling transduction remains ambiguous and necessitates additional investigation and clarification.

## 4 Primary cilia and reproductive system-related disorders

Primary cilia, resembling hair-like protrusions on the cell membrane, have emerged as focal points in recent investigations concerning tumorigenesis. Recent studies have elucidated that primary cilia serve as tumor suppressors in the majority of tumors, yet are diminished in tumor cells under specific pathological circumstances (Peixoto et al., 2020). Impairment of primary cilia structure and function under specific pathological conditions can precipitate a spectrum of ciliopathy-related disorders (Wiegering et al., 2021), encompassing Recurrent Implantation Failure (RIF), Epithelial Ovarian Cancer (EOC), Prostate Cancer (PCa), Breast Cancer, Bardet-Biedl Syndrome, and Joubert Syndrome.

### 4.1 Recurrent implantation failure (RIF)

Research has demonstrated the presence of primary cilia in human endometrial stromal cells. Within stromal cells, Sonic Hedgehog (SHH) stimulates the canonical Hedgehog (Hh) signaling pathway via primary cilia and facilitates decidualization through mechanisms implicating interleukin-11 (IL-11) and primary cilia (Li et al., 2023). Mounting evidence suggests that impaired decidualization is closely associated with Recurrent Implantation Failure (RIF). Aberrant primary cilia in the endometrium of patients with Recurrent Implantation Failure (RIF) play a pivotal role in human decidualization and can be modulated via the PTEN-PI3K-AKT-FOXO1 signaling pathway (Li et al., 2022).

### 4.2 Epithelial ovarian cancer (EOC)

Epithelial Ovarian Cancer (EOC) represents a heterogeneous neoplasm characterized by a high mortality rate attributed to challenges in early detection, constituting more than 90% of all ovarian malignancies (Zhang and Zhang, 2020; Pang and Chang, 2021). Studies suggest an association between Epithelial Ovarian Cancer (EOC) and aberrations in primary cilia formation. In ovarian cancer, the intraflagellar transport protein IFT20 is upregulated and modulates associated signaling pathways such as Hedgehog (Hh) and Platelet-Derived Growth Factor Receptor Alpha (PDGFRα) signaling (Egeberg et al., 2012; Deng et al., 2014). Hedgehog (Hh) signaling orchestrates cell proliferation and differentiation in numerous tissues during embryonic and fetal development. Numerous studies have recognized elevated Aurora A kinase activity and/or protein levels as prevalent characteristics of ovarian cancer (Egeberg et al., 2012).

### 4.3 Breast cancer

Breast cancer represents a prevalent endocrine disorder distinguished by elevated incidence and mortality rates. Research has revealed that genes associated with the formation and localization of primary cilia are downregulated in breast cancer cells, whereas oncogenes NEK2 and microtubule-depolymerizing kinase KIF24 are upregulated. Inhibition of these proteins can restore cilia formation and attenuate tumor cell proliferation ((Man et al., 2022)). Furthermore, transcription factors such as PTCH, SMO, and Gli, integral to the Hedgehog (Hh) pathway, modulate tumorigenesis. Nonetheless, breast cancer cells fail to activate downstream target genes of the Hedgehog (Hh) pathway via this pathway. In murine models of breast cancer, suppression of primary cilia exacerbates cancer cell invasion (Yang et al., 2020). Inhibition of primary cilia formation markedly upregulates the expression of downstream target genes of the Hedgehog (Hh) pathway in human breast cancer (Hassounah et al., 2017).

### 4.4 Prostate cancer (PCa)

Prostate cancer (PCa) stands as a prominent contributor to male cancer-related mortality on a global scale. Research has indicated the presence of primary cilia in stromal cells of the prostate, with a notable decrease observed in primary cilia abundance in prostate cancer tissue specimens relative to normal prostate tissue ((Mul et al., 2022)). Utilizing Wang Lin’s (Wang, 2021) gene knockout technique, it was demonstrated that depletion of TACC3 in prostate cancer cells elicits primary cilia formation, thereby partially reinstating ciliary abundance. TACC3 impedes primary cilia formation in prostate cancer (Wang, 2021). Primary cilia function to inhibit the Wnt signaling pathway in epithelial cells, and aberrations in ciliary structure may trigger activation of the Wnt signaling pathway in specific instances of prostate cancer (Hassounah et al., 2013). These findings imply that primary cilia dysfunction and heightened Wnt signaling are evident in certain types of human prostate cancer.

Despite their diminutive size, cilia intricately regulate a myriad of factors including Intraflagellar Transport (IFT) and signaling pathways, facilitating substantial information exchange within the cytoplasm. Hence, unraveling the molecular intricacies and regulatory dynamics underlying primary cilia formation and their repercussions on reproductive function holds the promise of not only deepening our comprehension of reproductive physiology but also opening novel vistas for mitigating and managing ciliopathies in clinical settings, thereby fostering greater protection of human health and wellbeing.

## 5 The role of primary cilia in reproductive physiology

A burgeoning corpus of research underscores the diverse functionalities of cilia across different segments of the reproductive tract, with profound impacts on the transport and fertilization processes of reproductive cells therein. Numerous reproductive tract disorders have been linked to aberrations in primary cilia, encompassing conditions such as ovarian cancer, breast cancer, prostate cancer, and cervical cancer (Legare et al., 2017). Concurrently, an intimate interconnection exists between primary cilia and reproductive physiology, with the Intraflagellar Transport (IFT) protein family, serving as pivotal constituents of primary cilia, being imperative for the preservation of reproductive wellbeing.

### 5.1 The male reproductive system

Within the male reproductive system, the epididymis, mirroring the structural composition of the fallopian tube with segments including the initial segment, head, body, and tail, possesses the capacity for sperm transportation (Girardet et al., 2019; Huang et al., 2022). Research findings have underscored the aggregation of primary cilia within the testes, epididymis, and prostate of mice, where they exert a pivotal role in sperm transport (Zhang et al., 2009; Ou et al., 2014; Bernet et al., 2018). Reports have indicated a potential association between primary cilia and male infertility (Ni et al., 2020). Database inquiries disclose predominant expression of the IFT20 gene in the testes, with IFT20 orchestrating the length regulation of primary cilia through its involvement in the Golgi apparatus. To substantiate the significance of IFT20 in preserving the normative functionality of cilia, Zhang Zhengang (Zhang et al., 2016) conducted a gene knockout experiment targeting the IFT20 gene in the reproductive cells of male mice, culminating in male infertility. To probe into the etiology of infertility, they conducted a comparative analysis of sperm morphology, quantity, and vitality between wild-type mice and IFT20 mutant mice. Their findings revealed a stark contrast wherein mutant mice exhibited significantly diminished sperm count, aberrant morphology, and compromised vitality in comparison to their wild-type counterparts, alongside a notable reduction in sperm count within the epididymis. Recent investigations have elucidated the role of COP9 signalosome subunit 5 (COPS5) as a prominent interacting partner of IFT20, jointly governing sperm development alongside IFT20 (122).

Moreover, deficiency of IFT25 in murine male germ cells precipitates a reduction in sperm count and impairs sperm morphology, characterized by rounded heads and truncated, curved tails (Huang et al., 2020). Primary cilia possess the capability to perceive and modulate the surrounding sperm environment and sperm functionality across various developmental stages of the male reproductive system via specialized mechanisms (diversified extension) (Huang et al., 2022). Literature documentation has elucidated that primary cilia located in immature testicular cells facilitate the formation of testicular tubules via the Hedgehog (Hh) signaling pathway (Dores et al., 2017; Sakib et al., 2019). Primary cilia within the epididymis are linked with undifferentiated columnar cells during the prepubertal phase and basal cells within the adult murine epididymal epithelium (Bernet et al., 2018). Basal cells within the epididymis contribute to epithelial regeneration via signaling mediated by primary cilia. Subsequent investigations have revealed that primary cilia located in mammary basal cells modulate the onset and progression of breast cancer through mediation of the Hedgehog (Hh) signaling pathway (Hassounah et al., 2017). Furthermore, absence of primary cilia was noted in principal cells within the epididymis, however, using transmission electron microscopy (TEM) and scanning electron microscopy (SEM), primary cilia were observed to protrude and elongate on the surface of castrated epididymal principal cells (Murakami et al., 1976). Hence, further exploration is warranted to elucidate the involvement of primary cilia in the development and maintenance of the epididymis.

### 5.2 The female reproductive system

It is widely acknowledged that decidualization of the endometrium holds pivotal significance in the onset of early pregnancy in females. Li Bo (Li et al., 2022) provided evidence for the presence of primary cilia on human endometrial stromal cells and their involvement in regulating decidualization via the PTEN-PI3K-AKT-FOXO1 signaling pathway. Recent investigations have uncovered the presence of primary cilia on trophoblast cells and early human placental tissues, which hold significant implications for human embryo implantation and placental development (Wang et al., 2017; Wang et al., 2019). Throughout embryo implantation, trophoblast cells originating from the blastocyst adhere to uterine wall stromal cells, subsequently undergoing differentiation to establish the placenta (Amack, 2022). Prior investigations have revealed that disruption of IFT88 results in ciliary loss. In order to assess the role of primary cilia in trophoblast cells, Wang (Wang et al., 2017) employed siRNA to suppress the expression of the IFT protein IFT88, leading to a reduction in both the number and length of cilia in cultured cells. Moreover, dysfunction of primary cilia in human placental mesenchymal stromal cells may precipitate threatened miscarriage (Romberg et al., 2022). McDermott (Mcdermott et al., 2010) further demonstrated the direct impact of primary cilia on the development of mammary gland branches. To validate the putative role of primary cilia in ovarian development, Johnson (Johnson et al., 2008) employed gene knockout technology to ablate the IFT88 gene in ovarian cells, resulting in ovarian dysfunction; findings suggested a potential involvement of IFT88 in modulating granulosa cell estrogen synthesis or secretion, consequently influencing ovarian function. Furthermore, additional studies have suggested a potential role for IFT88 in mammary stromal development, potentially impacting mammary gland development. Further investigation into the conditional loss of IFT88 in specific mammary gland cell types will aid in elucidating the precise mechanisms underlying the impact of IFT88 and cilia on ovarian function and mammary gland development.

Furthermore, Johnson (Johnson et al., 2008) employed a cell line expressing prx1-Cre (commonly utilized for gene deletion from early limb mesenchyme and cranial mesoderm) to target the disruption of IFT88. They observed Cre activity in cuboidal epithelial cells of immature ovarian follicles in newborn mice, which subsequently differentiated into granulosa cells during ovarian development. However, no cilia were detected, suggesting probable disruption of IFT88 in these newborn mice prior to puberty. Subsequent investigations revealed that prx1-Cre mutant mice displayed disruptions in the estrous cycle, abnormalities in ovulation, and delayed mammary gland development. Given the infertility observed in the mutant mice, histological techniques were employed to assess ovulation. Comparison of ovarian tissue sections from wild-type and mutant mice revealed the absence of corpora lutea in the ovaries of mutants, while numerous corpora lutea were observed in wild-type mice. These findings further substantiated the notion of impaired ovarian function in IFT88 mutant mice. In an attempt to stimulate corpus luteum formation, researchers induced superovulation in IFT88 mutant mice via exogenous hormone administration. However, mature oocytes were not observed, suggesting that oocyte maturation is predominantly reliant on the presence of primary cilia. Moreover, IFT88 mutant mice manifested delayed mammary gland development attributable to the absence of terminal bud formation, a process regulated by estrogen (Tasouri and Tucker, 2011). Future research endeavors may concentrate on investigating the impact of IFT88 deficiency on ovarian function and hormone regulatory mechanisms in murine models. Johnson (Johnson et al., 2008) further sought to investigate whether exogenous estradiol injection could rescue terminal bud development in IFT88 mutant mice. The findings demonstrated that estradiol injection facilitated the restoration of terminal bud development in mutant mouse mammary glands, suggesting that ovarian estrogen production is modulated by granulosa cell primary cilia; however, the precise regulatory mechanism remains elusive.

In conclusion, investigations into primary cilia in reproductive processes offer valuable insights for the diagnosis and management of reproductive disorders. The presence of primary cilia is paramount in both female and male reproductive processes. Primary cilia serve pivotal functions in sperm and epididymal development, oocyte viability, ovarian hormone secretion, and other physiological processes, thus contributing to the etiology and progression of reproductive disorders. Nonetheless, investigations into primary cilia predominantly concentrate on small animal models, particularly mice, and are primarily conducted at the cellular level, with limited research in large livestock species. A thorough and comprehensive exploration of the mechanisms underlying primary cilia function is anticipated to offer novel insights for enhancing animal reproductive efficiency and ameliorating human reproductive disorders.

## 6 Conclusion

Primary cilia, serving as pivotal organelles for intercellular signal transduction, exert indispensable roles in preserving normal physiological functions and modulating the onset and progression of reproductive system disorders. Primary cilia are intricately linked to a myriad of signaling pathways governing intercellular signal transduction, exerting significant influence on embryonic development, cellular polarization, and proliferation. Despite some advancements in recent research endeavors, investigations into primary cilia within the reproductive system remain relatively sparse. Consequently, numerous inquiries remain to be elucidated in the exploration of primary cilia’s role in reproductive physiology and pathogenesis. For instance, certain investigations have revealed that specific concentrations of estrogen possess the capability to elongate primary cilia. This observation prompts inquiries into the potential significant relationship between primary cilia and diverse sex hormones. Can their effects be mediated through the hypothalamic-pituitary-gonadal axis (HPG)? Furthermore, the presence of primary cilia in granulosa cells of murine ovaries has been validated. Is there a potential interaction between these entities? Do alterations occur in the quantity, length, and spatial arrangement of primary cilia in the uterus and ovaries under pathological conditions? Do the diverse signaling pathways modulated by primary cilia intricately influence the maintenance of normal physiological functions and the pathogenesis of reproductive system disorders? Which signaling cascade serves as the predominant regulatory mechanism? What are the specific mechanisms through which it mediates its effects? Could primary cilia potentially serve as novel targets for diagnosing or treating reproductive system tumors in the future? In conclusion, thorough investigations into the formation of primary cilia and their assorted signaling pathways in the reproductive system hold profound significance in elucidating the impact of primary cilia and their signaling cascades on reproductive physiology and in devising strategies for the prevention and management of associated reproductive disorders.

Amidst the burgeoning exploration of the Hedgehog (Hh) and Wnt signaling pathways, the intricate architecture and functionality of primary cilia, along with their pivotal roles in mammalian reproductive development, have emerged as focal points of inquiry on a global scale. While certain investigations have indicated the pivotal involvement of the Hh and non-canonical Wnt signaling pathways in governing the development of the female reproductive system, lingering debates persist regarding their interplay, alongside the elusive elucidation of the functions and mechanisms underpinning each stage of female reproductive development. Furthermore, the intricate interconnections between signals governing cell proliferation, polarity, reproductive development, and primary cilia, mediated by pathways such as Notch and TGF-β, remain incompletely delineated, while the intricate interplay between the Hh signaling pathway and other signaling cascades continues to evade full comprehension. Hence, there arises a pressing need for further inquiry to unravel the intricate mechanisms governing primary cilia and associated signaling pathways in reproductive development, thereby paving the way for a more profound comprehension of the underlying mechanisms implicated in reproductive system disorders stemming from primary cilia aberrations.
